# Purification of the recombinant green fluorescent protein from tobacco plants using alcohol/salt aqueous two-phase system and hydrophobic interaction chromatography

**DOI:** 10.1186/s12896-019-0590-y

**Published:** 2019-12-09

**Authors:** Jie Dong, Xiangzhen Ding, Sheng Wang

**Affiliations:** 1 Key Laboratory of Ministry of Education for Protection and Utilization of Special Biological Resources in the Western China, Yinchuan, 750021 People’s Republic of China; 2 Key Laboratory of Modern Molecular Breeding for Dominant and Special Crops in Ningxia, Yinchuan, 750021 People’s Republic of China; 3 School of Life Science, Ningxia University, 539 W. Helanshan Road, Yinchuan, Ningxia 750021 People’s Republic of China

**Keywords:** Green fluorescent protein, Plant virus, Transient gene expression, Aqueous two-phase system, Hydrophobic interaction chromatography

## Abstract

**Background:**

The green fluorescent protein (GFP) has been regarded as a valuable tool and widely applied as a biomarker in medical applications and diagnostics. A cost-efficient upstream expression system and an inexpensive downstream purification process will meet the demands of the GFP protein with high-purity.

**Results:**

The recombinant GFP was transiently expressed in an active form in agoinoculated *Nicotiana benthamiana* leaves by using *Tobacco mosaic virus* (TMV) RNA-based overexpression vector (TRBO). The yield of recombinant GFP was up to ~ 60% of total soluble proteins (TSP). Purification of recombinant GFP from the clarified lysate of *N. benthaniana* leaves was achieved by using an alcohol/salt aqueous two-phase system (ATPS) and following with a further hydrophobic interaction chromatography (HIC). The purification process takes only ~ 4 h and can recover 34.1% of the protein. The purity of purified GFP was more than 95% and there were no changes in its spectroscopic characteristics.

**Conclusions:**

The strategy described here combines the advantages of both the economy and efficiency of plant virus-based expression platform and the simplicity and rapidity of environmentally friendly alcohol/salt ATPS. It has a considerable potential for the development of a cost-efficient alternative for production of recombinant GFP.

## Background

Green fluorescent protein (GFP) was originally derived from jellyfish *Aequorea victoria* species, which exhibit an intensely natural fluorescence [1]. GFP has been regarded as a valuable tool in the field of biology and biotechnology [2]. Due to its widespread application as a molecular biomarker [3, 4], there is an increase in the demand for GFP with high-purity.

Through the application of DNA recombinant technology, GFP has successfully been produced by a variety of hosts [5]. Currently, the commercially available GFP produced by *Escherichia coli* costs approximately US$ 2000.00 per mg [6]. A cost-efficient upstream expression system and an inexpensive downstream purification process will be able to reduce the production costs and thereby meet the demands of the GFP with high-purity. Plants have been regarded as excellent biofactories for producing recombinant proteins of interest for research, pharma and industry [7]. It was estimated that proteins can be produced in plants at a cost of 10–50 fold less than in *Escherichia coli* [8]. Virus-based expression system can express the target proteins in plants at an extremely high level because of viral amplification [9]. In addition, plant platform offers an eco-friendly way to produce recombinant proteins largely due to low energy requirements and CO_2_ emission [10].

In order to achieve a high level of purity, diverse chromatographic techniques have been used to purify the recombinant GFP. In general, these chromatographic methods involve multistep, time-consuming and complicated operations, resulting in a higher purification cost [5]. Thus, an inexpensive method for GFP purification is highly needed. Aqueous two-phase system (ATPS) has been widely regarded as an alternative way for the separation and purification of proteins and other biomolecules [11]. Significant efforts have been made to develop different type of ATPSs and their applications in purification of various biomaterials [12]. Alcohol/salt ATPS is one of the promising members of the ATPS family [13]. The advantages of alcohol/salt ATPS include low cost, fast phase separation, simple operational procedures and easy scale-up [14]. Furthermore, this type of ATPS has an environmental friendliness aspect as ethanol and salt can be recycled via conventional processes [15].

Considering the excellent capabilities of plant viral expression vector and alcohol/salt ATPS, this work aimed to develop a cost-effective alternative for production of recombinant GFP. Plant viral amplicon-based gene expression system [16] was employed to transiently express recombinant GFP in *Nicotiana benthamiana* leaves by agroinfiltration. Subsequently, purification of GFP was achieved by combining an alcohol/salt ATPS stage with a further hydrophobic interaction chromatography (HIC) step. The GFP extraction efficiencies of each step were determined, and their purification aptitudes were evaluated. The fluorescence characterization of purified GFP was measured by using both gel-based imaging and the spectrofluorometric method.

## Results

### Transient expression of recombinant GFP in *N. benthamiana* leaves

**Fig. 1:**
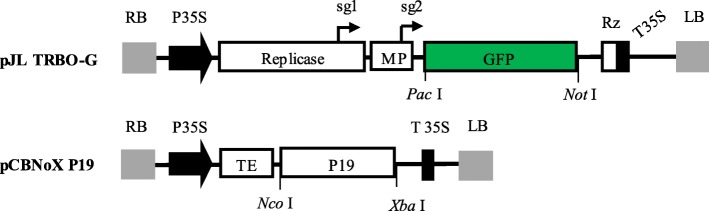
Structures of the expression vectors used in this study. pJL TRBO-G, TMV-based vectors used to express green fluorescent protein (GFP) in *N. benthamiana* leaves; RB, the right T-DNA border; P35S, duplicated *Cauliflower mosaic virus* (CaMV) 35S promoter; Replicase, RNA-dependent RNA polymerase of TMV; sg1 and sg2, subgenomic mRNA1 and mRNA2 promoter of the TMV; MP, movement protein of TMV; GFP, green fluorescent protein; Rz, ribozyme; T35S, CaMV polyA signal sequence/terminator; LB, the left T-DNA border; pCBNoX P19, RNA silencing suppressor expression vector used to co-infiltrate with pJL TRBO-G; TE, translational enhancer of *Tobacco etch virus* (TEV); P19, 19-kDa RNA silencing suppressor from TBSV; *Pac* I, *Not* I, *Nco* I and *Xba* I, restriction enzyme recognition sites

**Fig. 2:**
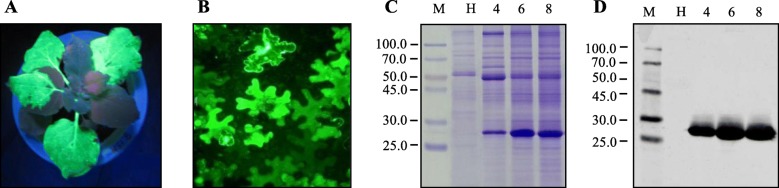
Analysis of accumulation of recombinant GFP in *N. benthamiana*. **a** Representative TMV vector-infiltrated *N. benthamiana* leaves under UV-illumination at 8 days post-inoculation (dpi). **b** Confocal microscopy image of GFP in infiltrated leaves. Green signal in image is due to GFP fluorescence. **c** Coomassie-stained polyacrylamide gel showing GFP accumulation in infiltrated *N. benthamiana* leaves. M, protein molecular weight marker in kDa; H, total soluble protein extracts from non-inoculated leaves, negative control; lane 4, 6 and 8, extracts from infiltrated leaves at 4, 6 and 8 dpi, respectively. **d** Western blot analysis of GFP from infiltrated *N. benthamiana* leaves. M, protein molecular weight marker in kDa; H, total soluble protein extracts from non-inoculated leaves, negative control; lane 4, 6 and 8, extracts from infiltrated leaves at 4, 6 and 8 dpi, respectively

The pJL TRBO-G vector (Fig. 1) was agoinoculated into *N. benthamiana* leaves in the presence of the suppressor of silencing P19. At 4–8 days after inoculation, high intensity of green fluorescence in the inoculated leaves was observed after illumination with long wave UV light (Fig. 2a). The cells exhibiting strong GFP signal could be seen in almost all cells in the agroinfected leaf area when examined under a fluorescence microscope (Fig. 2b). A protein corresponding to the expected molecular weight (27 kDa) was detected in the total soluble proteins extracted from the inoculated leaf tissues by both Coomassie stained polyacrylamide gel (Fig. 2c) and Western blot analysis (Fig. 2d). No signals were detected in samples from non-inoculated leaves (Fig. 2d). The GFP yield was up to ~ 60% of total soluble proteins (Table 1). All results together indicated that the recombinant GFP was successfully and efficiently expressed in the *N. benthamiana* leaves. The overexpression of GFP by plant amplicon-based vector provided a good foundation for the downstream purification of recombinant GFP.

**Table 1:** Quantitative specifications of the principal stages of GFP purification

Processing step	TSP^a^ (mg)	GFP^b^ (mg)	Purity^c^ (%)	GFP Yield ^d^ (%)
Extract	13.7	8.2	59.9	100.0
Ethanol extraction	5.5	4.8	87.3	58.5
*n*-butanol extraction	4.7	4.2	89.4	51.2
HiScreen Capto Butyl	2.9	2.8	96.6	34.1

^a^Amount of the TSP was determined according to BCA

^b^Amount of the GFP was measured by the ELISA

^c^Purity is defined as the amount of GFP divided by the amount of TSP in the same sample

^d^GFP Yield is defined as the amount of GFP recovered divided by initial amount of GFP in the crude extract of inoculated leaves

### Purification of GFP from *N. benthamiana* leaves

A procedure using alcohol/salt ATPS and HIC was applied for the isolation and purification of GFP from the inoculated *N. benthamiana* leaves.

**Fig. 3:**
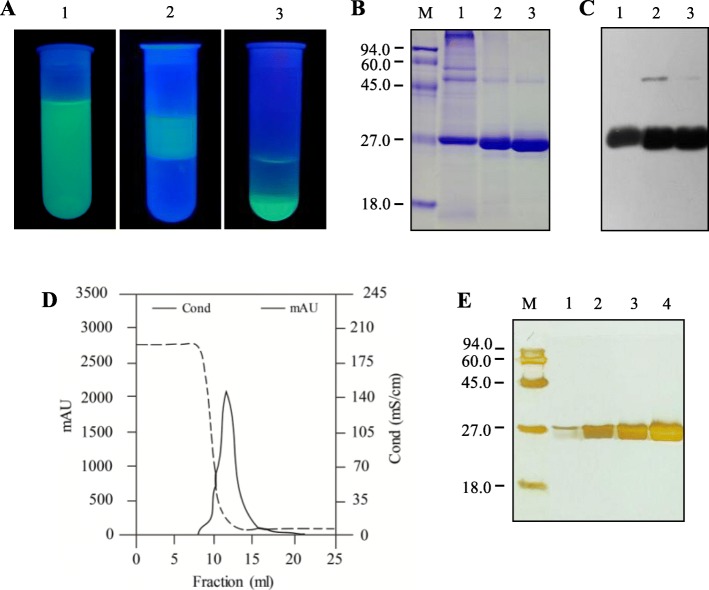
Purification of GFP by alcohol/salt ATPS and HIC. **a** Successive fractions in the course of alcohol/salt ATPS procedures under UV-illumination. 1, the supernatant of homogenate of infiltrated *N. benthamiana* leaves after centrifugation; 2, the phase separation showing GFP in the upper ethanol phase after ethanol extraction; 3**,** the phase separation showing GFP in the lower water phase after addition of *n*-butanol to the ethanol extract. **b** Analysis of the various GFP fractions from ATPS procedure by Coomassie-stained polyacrylamide gel. M, protein molecular weight marker in kDa; lanes 1, 2 and 3, the samples corresponding to the fractions from ATPS, as shown in a. **c** Immunoblot of the various GFP fractions in the course of ATPS. M, protein molecular weight marker in kDa; lanes 1, 2 and 3, the samples corresponding to the fractions in the course of ATPS, as shown in b. **d** Elution curves of the GFP purified by HIC. **e** Silver-stained polyacrylamide gel showing the HIC purified GFP. M, Protein molecular weight marker in kDa; lane1, 2, 3 and 4, twofold serial dilution of purified GFP

The alcohol/salt ATPS was performed by a two-step procedure. In the first step, the GFP was exclusively extracted into ethanol phase. The GFP fluorescence in the upper ethanol phase was clearly observed upon UV illumination after phase separation (Fig. 3a2). A thin layer of host cellular proteins was observed at the interphase (Fig. 3a2). In addition, analysis by Coomassie-stained polyacrylamide gel electrophoresis showed a reduction in plant proteins after ethanol extraction (Fig. 3b), indicating that GFP was partly purified by the step. The 27-kDa band was verified to be GFP by Western blot analysis (Fig. 3c). The purity of GFP was increased from 59.9% to about 87.3%, with a yield of 58.5% (Table 1). In the second stage of this proceedure, the GFP was recovered into the aqueous phase by addition of *n*-butanol, which is more hydrophobic than ethanol. After configuration, the two phases were separated, and GFP effectively partitioned into the lower aqueous phase (Fig. 3a3). The volume of aqueous phase decreased, indicating that the GFP solution was concentrated simultaneously, as verified by both the Coomassie-stained polyacrylamide gel (Fig. 3b) and Western blot (Fig. 3c). This step also provided modest additional purification, because the purity of GFP was raised from 87.3% to about 89.4%, with a yield of 51.2% (Table 1).

Because the aqueous phase may contain the residuals of organic solvents and salts, HIC chromatography was employed for their removal. A single peak was eluted (Fig. 3d) and silver-stained polyacrylamide gel analysis of purified recombinant GFP showed unique band of 27 kDa, even when high levels of GFP were examined (Fig. 3e). Overall purification resulted in GFP with purity above 95% and a yield of 34.1% (Table 1).

### The fluorescence characterization of purified GFP

Fluorescence characterization of purified GFP was carried out using both gel-based imaging [17] and conventional spectrofluorometer-based method.

**Fig. 4:**
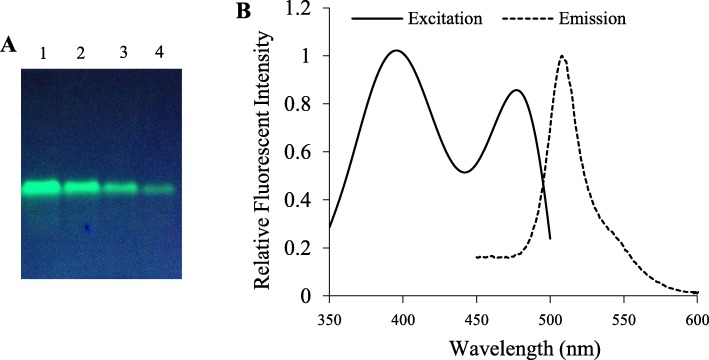
The fluorescence characterization of GFP purified from *N. benthamiana* plants. **a** Polyacrylamide gel was viewed under UV light. Purified GFP was mixed with SDS loading buffer (without reducing agent) and fractionated by electrophoresis through a polyacrylamide gel without prior heat denaturation, then photographed under UV light. Lane1, 2, 3 and 4, twofold serial dilution of purified GFP. **b** The fluorescence spectrum characterization of purified GFP. The maximum fluorescence excitation peak is at 396 nm; fluorescence emission peak is at 508 nm. Spectra were obtained at a concentration of 0.1 mg/ml and normalized to chromospheres absorption maxima

Because some kinds of alterations in GFP structure do not affect its chromophore fluorescence [18] and are not detectable using the spectrofluorometric method [17], gel-based imaging method, which is able to differentiate the alterations in GFP structure by relating the observed changes in the position of fluorescent bands, was employed to assess the structural changes of the purified GFP, which might take place during the purification process. GFP dilution samples, without heat treatment, were separated in a native discontinuous polyacrylamide gel. After electrophoresis, fluorescent image of GFP on the gel was captured using the Gel Doc XR (Bio-Rad) under UV illumination. The GFP bands were clearly visible in the gel under UV illumination, and no additional bands were observed (Fig. 4a), suggesting that the purification process did not cause detectable changes in GFP structure.

For spectrofluorometric analysis, samples were measured at the optimal excitation and emission wavelengths for GFP. Figure 4b shows the excitation and emission spectra of purified GFP from infected *N. benthamiana*. The emission spectrum shows a maximum at 505 nm and the excitation spectrum shows peaks at 395 nm and 470 nm (Fig. 4b). These spectroscopic properties are similar to those observed when GFP is purified by other methods.

## Discussion

Plants are an attractive alternative platform for the production of recombinant protein [19]. It offers numerous potential advantages; including low capital equipment, low energy requirements, easy scale-up, reduced risk of carrying pathogen contamination, and ability to post-translational modifications, etc. [9, 19]. Plant viral vectors are widely used as powerful tools for expressing heterologous proteins in plants with inexpensive production costs [20]. Here, we employed the viral expression vector to transiently express recombinant GFP in *N. benthamiana*, which is commonly used for producing target proteins by plant viral vectors [21]. Using TMV expression vector (pJL TRBO-G) with the help of RNA silencing suppressor, recombinant GFP in soluble form was expressed at an extremely high level (up to ~ 60% of TSP) in less than 1 week in the *N. benthamiana* leaves. In term of the yield, the plant-produced GFP is comparable with that obtained from *E. coli* (generally ranges from ~ 10% to ~ 50% of total protein). Moreover, it has been estimated that purification and downstream processing of recombinant proteins represents 80–90% of the cost of producing pharmaceuticals [22]. GFP could be produced in both soluble form and insoluble inclusion bodies from *E. coli* over-expression system, depending on culturing conditions (such as low growth temperatures, co-overexpression of molecular chaperones, etc.) [23, 24]. However, ~ 10% to ~ 20% of the recombinant GFP was found in the insoluble cell fraction even at optimal conditions [23]. Theoretically, the soluble form of plant-produced GFP may possess a good benefit for the cost reduction of the final GFP product. In addition, plant expression platform is more eco-friendly than the most of non-plant expression systems (such as bacteria, yeast, insect, and mammalian cell cultures, etc.). Altogether, we propose that the viral amplicon-based transient expression system is more suitable for the production of recombinant GFP than any other previously published method.

Currently, the various strategies, including chromatographic and non-chromatographic techniques, were developed to purify the recombinant GFP or its variants. Nevertheless, it is believed that the purification of GFP with the chromatographic methods generally involves multiple steps, time consuming, low throughput and of high cost [2]. In contrast, ATPS have been viewed as a potential alternative for protein purification because of its cost effectiveness and the simplicity of operation [5]. In addition, it is notable that alcohol/salt ATPS method is eco-friendly because ethanol and salt can be recycled. Although the yield of purified GFP could be considered modest in our study, alcohol/salt ATPS offers a considerably easier and faster way for purification of recombinant GFP. Otherwise, certain issues need to be considered while using alcohol/salt ATPS method in order to obtain effective GFP separation. Firstly, the soluble protein should be properly diluted before use, since GFP concentration exceeding 1.5 mg/ml can result in GFP co-precipitation with host proteins during phase separation. Secondly, operations should be performed at room temperature, because cooling can cause crystallization of ammonium sulfate. Lastly, exposure to ethanol can lead to a degree of protein denaturation. Thus, the step involving removing ethanol from the water-ethanol mixtures should be performed immediately.

The purpose of using HIC chromatography is initially to concentrate the sample and to remove the residuals of organic solvents and salts which may remain in the aqueous phase. However, we found that this step can further increase the GFP purity (from 89.4 to 96.6%). We speculate that some compounds, which may not be stained with coomassie dye, were removed by the HIC process. Moreover, we also observed that a little amount of fluorescent proteins were deposited on the top of the HIC column and they cannot be eluted even by low-salt buffer. Comparing with ATPS purified samples (Fig. 3b and c), a doubtful dimer GFP band was missed in the HIC purified sample (Fig. 3e), indicating that the HIC process probably also remove the oligomer version of recombinant GFP (soluble aggregates) [25]. One may argue that a single step of HIC can be used to purify GFP because it has advantage of handling large volume of samples and yielding a good result in terms of purity and yield [26]. Unfortunately, plant extracts and homogenates contain unique compounds, such as pigments, phenolics, and etc. [27]. Those compounds, especially phenolics, present obstacles in the downstream processes since they can interact with the target proteins during purification [27]. Moreover, the hydrophobic plant pigments can strongly bind to HIC media, thereby seriously reducing the useful life-time of the media to a few cycles of operation [28]. Otherwise, re-generation of the HIC media with high-frequency is costly since it requires the use of organic solvents [28]. Therefore, pretreatment is highly needed and a single step of HIC might be unattractive in practice for recovery and purification of recombinant proteins from plant tissues.

**Fig. 5:**
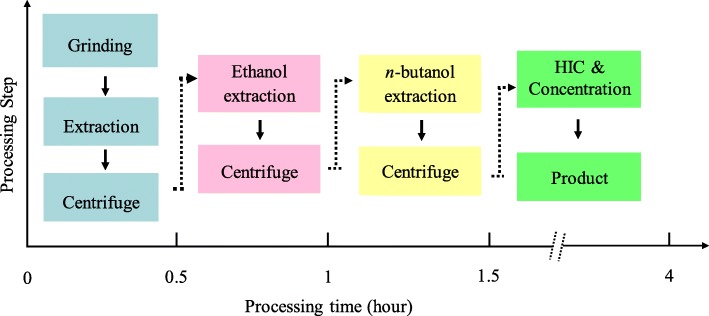
The flow diagram of the purification of GFP from *N. benthamiana* plants

In this study, a strategy for producing GFP, which combines advantages of both plant virus-based expression platform and alcohol/salt ATPS, was developed. The high level of recombinant GFP was achieved by plant virus-based overexpression vector and the high yield adds to the economy of the downstream process. Moreover, the complete purification process requires only a few steps, takes only ~ 4 h (Fig. 5) and recovers 34.1% of the protein, equivalent to a yield of ~ 1.7 g of GFP per kilogram of *N. benthaniana* leaves. The purity of final GFP product was determined to be more than 95%, and there were no changes in its spectroscopic characteristics. Although we did not experimentally compare our strategy with *E. coli*-based commercial strategy, two green technologies are involved in our strategy, thus making it more eco-friendly than what of the previously established methodologies. Accordingly, the developed expression and purification process in this study offer a cost-effective and concise alternative for the production of GFP.

## Conclusions

In summary, our study proposed a strategy for producing recombinant GFP. The procedures include overexpressing recombinant GFP in tobacco leaves via viral amplicon-based vectors and downstream purification by alcohol/salt ATPS and HIC. The strategy possesses cost-efficient and eco-friendly aspects and thereby has considerable potential for the development of an efficient process for large-scale production of recombinant GFP.

## Methods

### Plant materials and growth conditions

*N. benthamiana* seeds were kindly provided by Dr. Yongjiang Zhang of the Chinese Academy of Inspection and Quarantine (CAIQ). *N. benthamiana* seedlings were grown at 22–25 °C with 16 h dark period/8 h light period and 65% humidity in small 5 in. diameter plastic pots. All seedlings were watered on daily basis and supplemented with Hoagland solution once a week when required.

### Plasmids and *Agrobacterium* strains

The plasmids pJL TRBO-G, pCBNoX P19 and *Agrobacterium tumefaciens* GV3101 were kindly provided by Dr. John A. Lindbo of the Ohio State University. pJL TRBO-G, a *Tobacco mosaic virus* (TMV) RNA-based overexpression vector [16], was used to express GFP in *N. benthamiana* leaves using transient agroinfiltration. pCBNoX P19, expressing the 19-kDa silencing suppressor from *Tomato bushy stunt virus* (TBSV), was co-infiltrated with the pJL TRBO-G to prevent the RNAi-mediated gene silencing in plants. The plasmids were transformed into *A. tumefaciens* GV3101 using the freeze-thaw method.

### Agroinfiltration of *N. benthamiana* plants

*A. tumefaciens* GV3101 carrying either pJL TRBO-G or pCBNoX P19, were grown in 4 ml LB medium for 24 h at 28 °C and shaking at 250 rpm. The cultures were then transferred into 100 ml LB medium having 200 μM of acetosyringone (Sigma-Aldrich) grown overnight at 28 °C and shaking at 250 rpm. Cells were harvested by centrifugation at 3000 *g* for 10 min and re-suspended in infiltration buffer (pH 5.6, 10 mM MES, 10 mM MgCl_2_ and 200 μM acetosyringone) to achieve an OD_600_ of 0.4. The pJL TRBO-G expression vector was mixed in a 1:1 volume ratio with the gene-silencing suppressor (pCBNoX P19). The mixed *Agrobacterium* suspensions were incubated in the dark at room temperature for 2–3 h before infiltration. The incubated *Agrobacterium* suspensions were infiltrated into the abaxial surface of leaves using a 1-ml syringe without needle. The agroinfiltrated plants were incubated in the growth chamber for 4–8 days after which the leaves were harvested.

### GFP imaging

The GFP fluorescence was monitored by illumination with a hand-held long-wave UV source (UVP Blak-Ray 100AP) and was photographed with a Cannon G6 digital camera. For microscopic analysis, GFP-positive leaf cells were visualized using confocal laser scanning microscopy (Leica TCS STED Microscopy).

### Total soluble proteins (TSP) extraction and protein quantification

Protein samples were prepared by freezing agroinfiltrated leaves in liquid nitrogen and grinding to a fine powder with a mortar and pestle. The Extraction Buffer (50 mM Tris-HCl, pH 8.0, 150 mM NaCl, 10 mM EDTA) was added into the leaf powder at a ratio of 1: 4 (g/ml). Extracts were clarified by centrifugation at 12,000 g for 15 min at 4 °C. The supernatant containing TSP was recovered. Total protein content was determined by BCA protein assay kit (Pierce). The concentration of GFP was measured by GFP ELISA Kit (Abcam) according to the manufacturer’s specifications.

### Purification of GFP by alcohol/salt ATPS

0.3 ml of 5 M NaCl and 2.33 ml of saturated ammonium sulfate were added in turn to a 1-ml aliquot of TSP. Subsequently, the anhydrous ethanol was immediately added to the entire solution at a ratio of 1: 3 (volume-to-volume, v/v) and vigorously shaken for 30 s. The phases were separated by centrifugation at 3000 g for 5 min and the upper ethanol phase containing GFP was carefully collected. Afterward, *n*-butanol was added to the ethanol extracts at a 1: 4 volume-to-volume ratio. After shaking and centrifugation as described above, the lower aqueous phase containing GFP was recovered.

### Purification of GFP by HIC

AKTA Purifier system (GE Healthcare) equipped with an HIC column was used for further purification of GFP. Ammonium sulfate was added to aqueous phase obtained from the previous step up to the final concentration of 1.7 M. The solution was filtered through a 0.22 μm filter (Millipore) and loaded onto 4.7-ml HiScreen Capto Butyl column (GE Healthcare) equilibrated with Binding Buffer (10 mM Tris-HCl, pH 8.0, 10 mM EDTA, and 1.7 M ammonium sulfate). After extensive washing with the Binding Buffer, the GFP was eluted at 1 ml/min with Elution Buffer (10 mM Tris-HCl, pH 8.0, 10 mM EDTA).

### Sodium dodecyl sulfate-polyacrylamide gel electrophoresis (SDS-PAGE) and Western blot

Protein samples were subjected to 12% SDS-PAGE gels. After electrophoresis, gels were either stained with Coomassie brilliant blue or silver. For Western blot analysis, the proteins were transferred to a 0.45 μm nitrocellulose membrane (Sigma-Aldrich) using semi-dry electrophoresis transfer (Bio-Rad Trans-Blot SD system). The blot was developed with rabbit anti-GFP antiserum (Abcam) diluted 1:5000, followed by secondary goat anti-rabbit antibody conjugated with alkaline phosphatase (Abcam). Specific immunoreactive proteins were detected using a Western Blot ECL Plus kit (GE Healthcare).

### Gel-based imaging

Serial 2-fold dilutions of the purified GFP were prepared with double-distilled water. The diluted samples were mixed with SDS-PAGE gel-loading buffer (without reducing agent). A native and discontinuous polyacrylamide gel (PAGE) electrophoresis system was employed to fractionate the samples, with no prior heat treatment. Polyacrylamide gel with a 4% (w/v) of stacking gel and a 15% (w/v) of resolving gel were used in this study. After electrophoresis, the gel was captured using the Gel Doc XR (Bio-Rad).

### Fluorescence spectroscopy

Samples were diluted in 10 mM Tris-HCl, 10 mM EDTA, pH 8.0 buffers and fluorescence spectra were recorded on a Hitachi F-4500 spectrophotometer at room temperature. Excitation spectra were measured between 350 and 500 nm with the emission wavelength fixed at 508 nm. Emission spectra were measured between 450 and 600 nm with the excitation wavelength fixed at 396 nm.

## Data Availability

The datasets used and/or analysed in the current study are available from the corresponding author upon reasonable request.

## Abbreviations

*A. tumefaciens*: *Agrobacterium tumefaciens*
ATPS: Aqueous two-phase system
CaMV: *Cauliflower mosaic virus*
GFP: Green fluorescent protein
HIC: Hydrophobic interaction chromatography
kDa: kilo-Dalton
*N. benthamiana*: *Nicotiana benthamiana*
rpm: rotation per minute
SDS-PAGE: Sodium dodecyl sulfate-polyacrylamide gel electrophoresis
TBSV: *Tomato bushy stunt virus*
TEV: *Tobacco etch virus*
TMV: *Tobacco mosaic virus*
TSP: Total soluble proteins
UV: Ultra-violet
